# Simultaneous and rapid detection of avian respiratory diseases of small poultry using multiplex reverse transcription-Polymerase Chain Reaction assay

**DOI:** 10.1016/j.psj.2023.102852

**Published:** 2023-06-08

**Authors:** Tohid Piri-Gharaghie, Ghazal Ghajari, Naz Tavakoli Lahijani, Renzon Daniel Cosme Pecho, Fahdil Hussam, Roxana Yolanda Castillo-Acobo, Mona Aghassizadeh-Sherbaf

**Affiliations:** ⁎ Biotechnology Research Center, Shahrekord Branch, Islamic Azad University, Shahrekord, Iran; Department of Biology, Faculty of Biological Sciences, East Tehran Branch, Islamic Azad University, Tehran, Iran; † Department of Cell and Molecular Biology, Faculty of Biological Sciences, Kharazmi University, Tehran, Iran; ‡ Faculty of Pharmacy, Tehran University of Medical Sciences, Tehran, Iran; § Department of Biochemistry, Universidad San Ignacio De Loyola (USIL), Lima, Peru; # Medical Technical College, Al-Farahidi University, Baghdad, Iraq; ǁ Universidad Nacional de San Agustin de Arequipa, Arequipa, Peru; ¶ Department of Biology, Faculty of Basic Sciences, Islamic Azad University, Tehran East Branch, Tehran, Iran

**Keywords:** multiplex-PCR assay, poultry, virus, clinical detection

## Abstract

Major viral infections, such as Newcastle disease virus, infectious bronchitis virus, avian influenza virus, and infectious bursal disease virus, inflict significant injury to small poultry and tremendous economic damage to the poultry sector. This research aims to develop a multiplex reverse transcriptase polymerase chain reaction (**m-RT-PCR**) approach to simultaneously determine these important viral pathogens. The conserved segment of various viral genetic sequences was used to design and synthesize specific primers. Moreover, as positive controls, recombinant vectors were synthesized in this investigation. The d-optimal approach was used to improve PCR conditions in this investigation. Positive controls and clinical samples were used to assess the m-PCR assay's specificity, sensitivity, repeatability, and reproducibility. According to the sensitivity test findings, the m-PCR technique could generate the 8 target genes from viral genomes using 1 × 102. In addition, 8 viral pathogens were detected from the infected samples. The findings also suggest that live animal oral swabs were not significantly different from tissue sampling of a dead animal (*P* < 0.05), and this kit had a high sensitivity for analyzing both types of samples. The suggested m-PCR test may detect and evaluate viral infection in birds with excellent specificity, sensitivity, and throughput.

## INTRODUCTION

Avian influenza virus (**AIV**) (Wen et al., 2022), Newcastle disease virus (**NDV**) (Ul-Rahman et al., 2022), infectious bronchitis virus (**IBV**) (Shariatmadari, 2000; Birhanu et al., 2022), and infectious bursal disease virus (**IBDV**) (Beiranvand et al., 2022; Hu et al., 2022) are the principal viruses that induce serious financial losses in the small poultry sector (Yao et al., 2019). AIV has been derived from many small poultry species. Small poultry is often the major reservoir host. On the other hand, highly virulent avian influenza has the potential to cause substantial fatality in small poultry (de Camargo et al., 2022). As a result, detecting AIV in small poultry is critical in epidemiological studies (de Camargo et al., 2022). Small poultry is typically regarded as possible reservoirs for NDV, like AIV, which has been detected in Iran occasionally. Furthermore, NDV was common in small poultry across Iran, resulting in significant economic losses (Sabouri et al., 2018).Infectious bursal disease (**IBD**), commonly known as Gumboro syndrome, is a viral infection in the Avibirnavirus species (family Birnaviridae). While geese, waterfowl, guinea hens, birds, and ostriches can be infected, the symptomatic illness only develops in poultry. Only chicks under the age of 10 wk are often clinically impacted. Clinical indications are frequently absent in older hens (Dey et al., 2019).

Despite substantial immunization, the causal agent of IBDV is an extremely infectious and inflammatory illness of chickens that causes enormous economic losses to the poultry sector (Wang et al., 2021). An avian coronavirus, an enclosed single-stranded RNA virus with a distinctive spike-like protrusion on the outside of its membrane, causes IBV. Mutation in the viral spike molecule results in the emergence of various strains of the virus, which might also differ locally. The pathogen spreads quickly throughout the flock, causing respiratory discomfort (Stevenson-Leggett et al., 2021). In simple infections, fatality is normally minimal; nevertheless, some virus variants have a preference for the renal, which causes death due to renal disease. Consequences, including coinfection with other diseases, may also contribute to increased fatality (Lin and Chen, 2017). IBV is ubiquitous in all nations with a large chicken economy, with infection rates surpassing 100% in most places (Piri Gharaghie et al., 2021).

Given the significant threat posed by these infections to the small poultry sector, quick and simple approaches for identifying these pathogens and applying preventative actions to decrease financial damage as soon as feasible (Wang et al., 2017; Yang et al., 2017) are critical. Viral diagnostic tools include included virus isolation and characterization, serological diagnosis, immuno-electron spectroscopy, enzyme-linked immune sorbent assay (**ELISA**), lateral flow assay (**LFA**), and polymerase chain reaction (**PCR**). Moreover, the procedure is time demanding, which limits its applicability in immediate rapid diagnosis (Xu et al., 2012). Immunoassay-based approaches, such as ELISA, are commonly utilized. The issue with this technology is that it requires specific antibodies, which are time demanding and tiring to produce (Smith and Dunstan, 1993). Immuno-electron scanning necessitates specialized equipment and a large volume of the virus, making it unsuitable for diagnostic techniques (Mirmajlessi et al., 2015). In contrast to these approaches, PCR is a widely utilized technique in molecular biology (Hao et al., 2016). It can multiply a single copy or a few copies of a given DNA sequence dramatically. Because of its high sensitivity, nonstrict detecting requirements, great specificity, fast response, and reliability, it has been widely employed in diagnostic medical research for a wide range of pathogen identification (Almeida et al., 2017; Diao et al., 2021). M-PCR (m-PCR) relates to PCR amplification that employs 3 or more specific primers in a PCR reaction volume to amplify several genomic sequences at the same time (Chen et al., 2013). M-PCR has unrivaled benefits over uniplex PCR, notably high replication accuracy, time savings, and maximum throughput (Ali et al., 2015; Piri Gharaghie et al., 2021). More notably, this technique can differentiate between many viruses at the same time; it is an efficient way for quick identification of mixed-virus disease in early diagnosis (Moustacas et al., 2013; Cassedy et al., 2021). The goal of this study was to design and develop an m-RT-PCR technology capable of identifying and distinguishing significant serotypes of 4 main small poultry virus infections: AIV, NDV, IBV, and IBDV.

## MATERIALS AND METHODS

### Declaration of Ethics

This experiment was carried out in compliance in accordance with ARRIVE guidelines (https://arriveguidelines.org/arrive-guidelines). Autoclave cotton swabs were used to carefully gather biological specimens from normal poultry. The chickens were not anesthetized before testing, and following the sample, they were monitored for 30 min before being transferred to their cages.

### Viral Variants and Growing Conditions

Pathogenic viruses, including AIV, NDV, IBV, and IBDV subtypes, were collected from infected animals (Table 1). The viruses were stored in the AmitisGen Tech Dev Group and Parsian BioProducts companies. All medical swab specimens were tested from healthy poultry's cloacae, larynges, and tracheae. Also, the genome of the viruses was prepared from the Razi Serum and Vaccine Institute (Karaj, Iran). The genome of the viruses was stored in AmitisGen Tech Dev Group Parsian BioProducts companies.

**Table 1:** Viral pathogens used in this research.

Pathogens	Field samples	Description								
		M	H1	H2	H3	H5	H6	H7	H9	H10
Avian influenza virus (AIV)	H9N9, H9N1, H9N2,…(Species for H9N1….10)	+	-	-	-	-	-	-	+	-
	AIV H1N1 Human/NJ/8/76	+	+	-	-	-	-	-	-	-
	AIV H2N3 Duck/HK/77/76	+	-	+	-	-	-	-	-	-
	AIV H3N6 AIV Duck/HK/526/79/2B	+	-	-	+	-	-	-	-	-
	AIV H3N2 A/Chicken/Guangxi/015C10/2009	+	-	-	+	-	-	-	-	-
	AIV H3N2 A/Duck/Guangxi/015D2/2009	+	-	-	+	-	-	-	-	-
	AIV H3N6 A/pigeon/Guangxi/020P/2009	+	-	-	+	-	-	-	-	-
	AIV H3N6 A/Duck/Guangxi/175D12/2014	+	-	-	+	-	-	-	-	-
	Inactivated H5N1 AIV Re-1	+	-	-	-	+	-	-	-	-
	cDNA of H5N3 AIV Duck/HK/313/78	+	-	-	-	+	-	-	-	-
	cDNA of AIV H5N2/chicken/QT35/87	+	-	-	-	+	-	-	-	-
	cDNA of AIV H5N5/chicken/QT35/98	+	-	-	-	+	-	-	-	-
	cDNA of AIV H5N7 A/waterfowl/GA/269452-56/03	+	-	-	-	+	-	-	-	-
Newcastle disease virus (NDV)	NDV- Class I	Newcastle disease virus strain Lasota (isolated form avian)								
	NDV- Class II	Newcastle disease virus								
infectious bronchitis virus	IBV-CK/CH/HUN/20180415	Preserved in laboratory (isolated from chicken)								
	IBV-CK/MEX/2725/21	Preserved in laboratory (isolated from chicken)								
Infectious bursal disease virus (IBDV)	IBDV(EGY-CK-IBDV-DAKH88-2021-VP2 VP2 gene, FJ20-9407)	Preserved in laboratory (isolated from chicken)								
	IBDV(IR/H2965-17/18 VP2 gene,TN46/19)	Preserved in laboratory (isolated from chicken)								

### Extracting Nucleic Acids

The nucleic acid of the viral pathogens was isolated and diluted in a nuclease-free solution by using the Viral RNA/DNA Extraction Kit (Takara Bio, Japan). The Reverse Transcription Kit was used to convert the RNAs of AIV, NDV, IBV, and IBDV into cDNA (Thermo Scientific, Waltham, Massachusetts, US). Spectrophotometry was used to assess the quantity and quality of each genome (Thermo Scientific). The cDNA was kept at a temperature of −20°C.

### Primer Designing for Viral Genomes

The AIV, NDV, IBV, and IBDV strains’ whole-genome sequences were obtained from GenBank, and DNAMAN was used to align the conserved domain of viral-specific genes (LynnonBiosoft). Using oligo7 (https://www.oligo.net/), 2 pairs of particular primers for each virus based on the sequence alignment results were designed. Macrogen, Inc. produced the primers indicated in Table 2 (Macrogen, Seoul, South Korea).

**Table 2:** List of primers used in this study.

Disease	Name	Gene/GenBank	Seq	TM (°C)	Size
Newcastle disease virus (NDV)	NDV- Class I	MZ737127.1	F: ATGGATCCCAAGCCTTCTAC	57	433
			R: TGGCTTGTATGAGGGCAGAA		
	NDV- Class II	JX193770.1	F: ATGGGCYCCAGACYCTTCTAC	57	535
			R: CTGCCACTGCTAGTTGTGATAATCC		
Avian influenza virus (AIV)	(Multispecies) H5N1, H5N3, H5N8,…	EU443579.1	F: TGTACGGACTTGCTGTGGCC	57	106
			R: GAGACTGAAGACCTGGCTGTT		
	H9N1, H9N2, H9N3,…	KT368793.1	F: ATCGGCTGTTAATGGAATGTGTT	57	221
			R: TGGGCGTCTTGAATAGGGTAA		
Infectious bronchitis virus	IBV CK/MEX/2725/21	EF382355.1	F: TGGTTGGCATTTACACGGGG	57	228
			R: CAATGGGTAACAAACAC		
	IBV-CK/CH/HUN/20180415	MN509338.1	F: GGCAATTCTACATCTG	57	446
			R: AGATGTATCTAAAATAGC		
Infectious bursal disease virus (IBDV)	IBDV(EGY-CK-IBDV-DAKH88-2021-VP2 VP2 gene, FJ20-9407 )	X03993.1	F: CAACAGTGTAGTCTCTCCCG	57	150
			R: GATGTTTGCTGTCATTGAA		
	IBDV(IR/H2965-17/18 VP2 gene,TN46/19)	MW316417.1	F: TAGTTGCCACCGTGGATCG	57	350
			R: CAATCACACTGTTCTCAGCC		

### Preparation of Standard Plasmid

In this research, 4 recombinant vectors were employed and were constructed as positive controls. To acquire the recombinant plasmids pcDNA3.1(+)/AIV, pcDNA3.1(+)/NDV, pcDNA3.1(+)/IBV, and pcDNA3.1(+)/IBDV, particular target segments were first generated using the primers (Table 1), and then these sequences were introduced into the pcDNA3.1(+) plasmid (Shenzhen, China). The vector copy number was determined by using the following equation: copy number (copies/μL) =NA (copies/mol) concentration (g/μL)/ MW (g/mol), where NA is Avogadro's number and MW is the reference times 340 (https://www.technologynetworks.com/tn/tools/copynumbercalculator) (Gao et al., 2014).

### Reverse Transcription-PCR Assay

Each reaction mixture had an overall volume of 25 μL, including 2.5 μL 10× Buffer (Mg2+ free), 4 μL (25 mM) MgCl_2_, 0.75 μL dNTP (10 mM each), 0.25 L (5 U/μL Taq DNA Polymerase Vazyme, China), 1 μL forward primer, 1 μL reverse primer, 2 μL single-virus vector, and 13.5 μL ddH_2_O. The PCR procedure was performed as follows: Predenaturation at 95°C for 5 min, followed by denatured nucleic acids at 95°C for 60 s, annealing at 55°C for 40 s, elongation at 72°C for 45 s, 35 cycles, and a final extension at 72°C for 10 min. About 1.5% agarose gel electrophoresis was used to evaluate the PCR results. Double distilled water was used as the blank control.

### Multiplex Reverse Transcription-PCR Assay: Experimental Design

A D-optimal strategy of 25 trials was used to optimize the m-PCR procedure. Annealing rates (49°C–67°C), Mg2+ ratios (1–6 mM), Taq DNA Polymerase ratios (0.02–0.06 U/L), and dNTP concentrations (0.08–0.48 mM) were all taken into account. In consequence, the data analysis was determined by the intensity of the PCR-produced bands. MODDE 12.1 program was used for all evaluations (Umetrics, Sweden).

### Sensitivity and Specificity Analysis of the **m-PCR** Approach

a) Specificity analysis of the m-PCR: Primer blast analysis was used to assess the specificity of m-PCR. The combined vectors were diluted from 1 × 10^6^ to 1 × 10° copies/µL using a 10-fold gradient diluting procedure to test the m-PCR technique's sensitivity. Also, *Escherichia coli, Salmonella*, and *Clostridium perfringens* were used to test the specificity of the m-PCR approach.
b) The m-PCR technique's efficiency: By using a 10-fold gradient dilution method, each one of the recombinant DNA vectors was diluted from 1 × 10^6^ to 1 × 10^0^ copies/μL. After that, m-PCR was carried out to determine limit of detection (**LOD**). m-RT-PCR reaction was performed for each sample with 3 replications.
c) Detection limit, analytic sensitivity, and normal range: Two inactive positive samples of NDV, AIV, IBV, and IBDV viruses at a concentration of 1 × 10^6^ copies/µL were evaluated at the Virology Research Center to verify the technique. Each sample received 3 replications of the m-RT-PCR procedure.

### Repeatability and Reproducibility Analysis of the **m-PCR**

a) Evaluation of reproducibility within a single LOT: On the same day, the technique's repeatability was determined. Two users evaluated 20 positive control (1 × 10^2^ copies/µL recombinant plasmids) using kits made by a single LOT. The correlating percentages of different LOTs were evaluated.
b) Evaluation of reproducibility between multiple LOTs: The evaluation of reproducibility was carried out on 2 different LOTs. Kits manufactured in 2 LOTs were used to examine 20 recombinant plasmids. The recombinant plasmid amounts were 1 × 10^2^ copies/µL.

### Kit Validation Test

To validate the kits synthesized in this study, a multiplex kit for respiratory diseases (GeneProof, Czech Republic, CAS No. QAV054134) was prepared. In 3 different laboratories from different parts of Tehran, multiplex-PCR test was performed on recombinant plasmids at the concentration of 1 × 10^2^ copies/µL. The results obtained from the kit synthesized in this study were compared with the results of the standard kit (GeneProof, Czech Republic). The test was repeated 3 times.

### Simulation of Coinfection and Identification of Clinical Specimens

The coinfection investigation aimed to see if the m-PCR approach was practicable. In addition, 200 clinical samples (100 tissue specimens and 100 oral swabs) were gathered from small poultry farms and live chicken markets (these specimens were acquired with the permission of the animals’ owners, and the animals’ suffering was reduced). For PCR amplification, the standard m-PCR technique was applied. Uniplex PCR and conventional or published PCR procedures were used to corroborate the results. The positive PCR amplification products were then sequenced to corroborate the detection rate.

### Statistical Analysis

GraphPad Prism 5.0 was used to examine the data and perform statistical tests. A one-way ANOVA was used to compare means, followed by a Tukey–Kramer post hoc test with a 95% confidence interval. Differences were considered significant at *P* < 0.05.

## RESULTS

### The m-PCR Technique Was Optimized and Established

With 20 runs completed in one randomly selected LOT, a D-optimal technique was chosen to optimize the m-PCR process. With annealing temperature at 57°C, Mg2+ concentration at 4 mM, Taq DNA Polymerase concentration at 0.05 U/L, and dNTP concentration at 0.32 mM, we reached the final optimal settings, taking into account the economic approach, with the required compromise. We developed the m-PCR technique, which could efficiently generate duplex and triplex genes, using the ultimate ideal primers and settings. The primers’ BLAST findings are presented in Table 3. The blast primer results of this study showed that each primer corresponds to different serotypes of the target viruses.

**Table 3:** The BLAST results of primers used in this study.

Name of virus	Name of primers	The results of BLAST	Identity ratios	E value
NDV	NDV- Class I	13 type of Avian orthoavulavirus 1 strain	100%	1.2
		87 type of Newcastle disease virus class 1	100%	1.2
	NDV- Class II	6 type of Newcastle disease virus class 2	100%	1.2
AIV	Multispecies	Influenza A virus H1… H12	100%	1.2
	H9- species	Influenza A virus H9N1…10	100%	0.020
IBV	IBV CK/MEX/2725/21	IBV multispecies1	100%	1.2
	IBV-CK/CH/HUN/20180415	IBV- multispecies2	100%	0.32
IBDV	IBDV(EGY-CK-IBDV-DAKH88-2021-VP2 VP2 gene, FJ20-9407)	Infectious bursal disease virus gene for polyprotein	100%	1.2
	IBDV(IR/H2965-17/18 VP2 gene,TN46/19)	IBDV multispecies	100%	4.9

### Construction and Identification of Recombinant Plasmids

**Figure 1:**
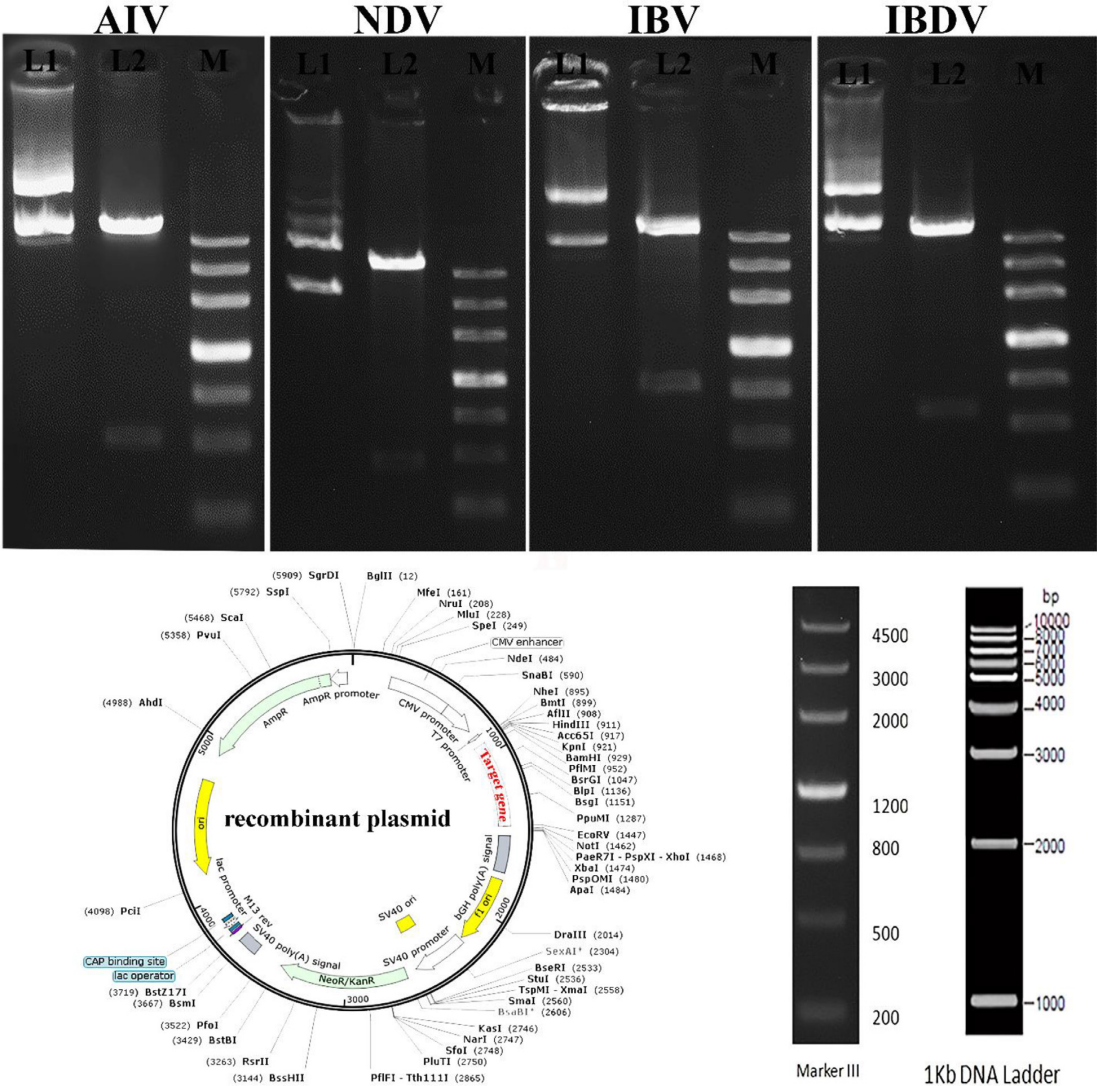
Identification of recombinant plasmids. pcDNA3.1(+)/AIV, pcDNA3.1(+)/NDV, pcDNA3.1(+)/IBV, and pcDNA3.1(+)/IBDV are shown schematically. BamHI and EcoRV sites were used to introduce the genes into pcDNA3.1(+). Electrophoresis was used to separate the DNA plasmids. Lane 1: empty pcDNA3.1(+); Lane 2: recombinant plasmids; Lane M: DNA markerIII. Gels are cropped to increase clarity and improve presentation conciseness. Samples were obtained from this experiment and the gels were processed in parallel. (10.6084/m9.figshare.20092595)

Virulence genes of AIV, NDV, IBV, and IBDV strains were cloned into the eukaryotic expression vector pcDNA3.1(+), as shown in Figure 1, separately. DNA sequencing revealed that the gene sequences from the 4 recombinant plasmids were identical to the AIV, NDV, IBV, and IBDV strains. BamHI and EcoRV were used to digest the plasmids that had been constructed. The digestion products separated electrophoretically at 520 and 5400 bp for AIV, 470 and 5400 bp for NDV, 840 and 5400 bp for IBV, and 600 and 5400 bp for IBDV strains (Figure 1), indicating that the recombinant plasmid was successfully constructed.

### The m-PCR Approach Has a High Level of Specificity

**Figure 2:**
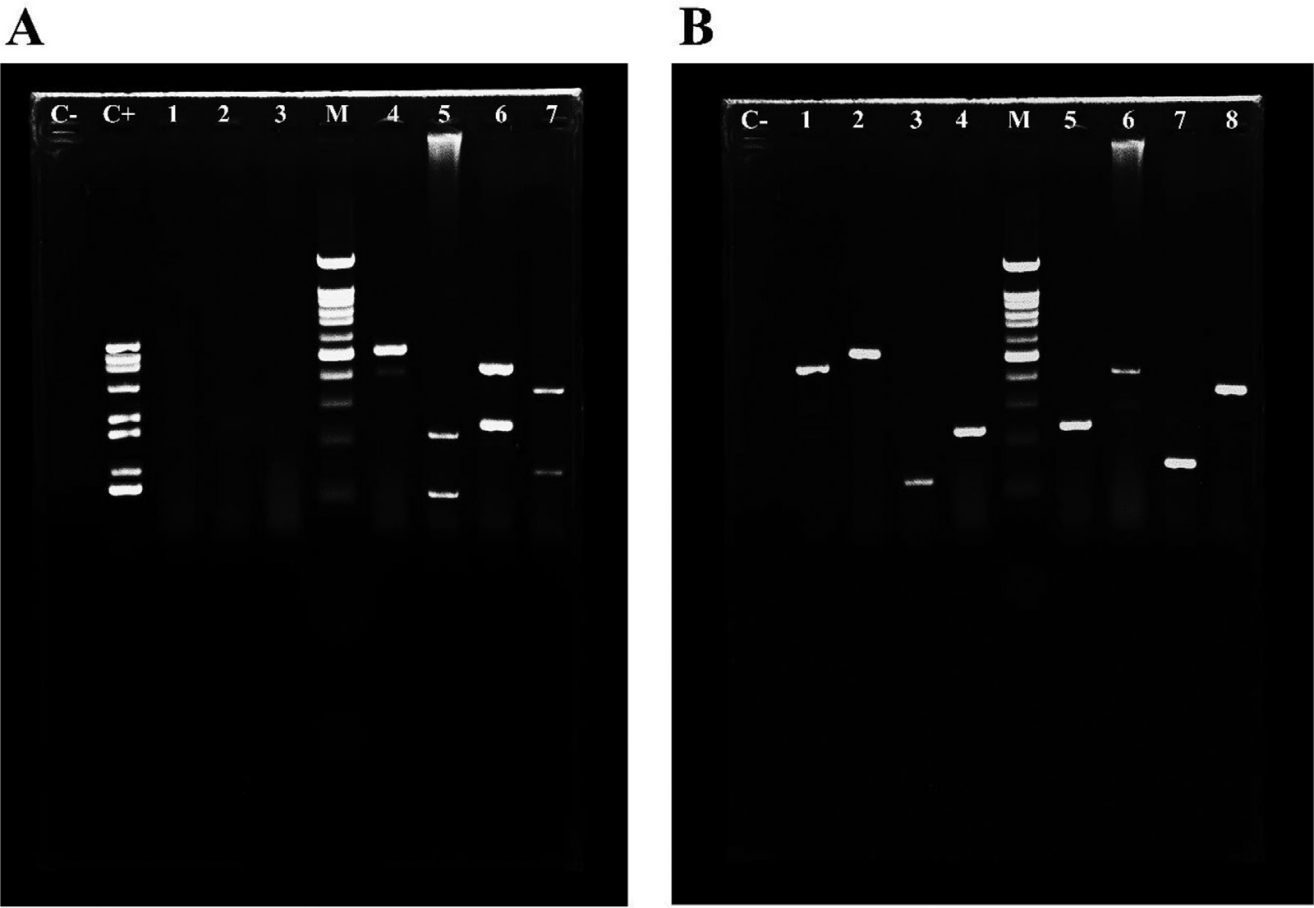
(A) Specificity of the m-PCR method with recombinant plasmids and other pathogens; Lane 1: *Escherichia coli*, Lane 2: *Salmonella*, Lane 3: *Clostridium perfringens*, Lane 4: NDV-Class I and NDV-Class II, Lane 5: AIV, Lane 6: IBV, Lane 7: IBDV. M: 100 bp marker; C−: negative control; and C+: the pool of recombinant plasmids as the positive control. (B) Specificity of the KIT in uniplex-PCR method. Lane 1,2: AIV, Lane 3,4: NDV, Lane 5,6: IBV and Lane 7, 8: IBDV and M: 100 bp marker; C−: negative control. Samples were obtained from this experiment and the gels were processed in parallel. (10.6084/m9.figshare.20092559)

These 8 viruses, as well as *E. coli, Salmonella*, and *C. perfringens*, were used to test the specificity of the m-PCR approach. The band for each viral pathogen was apparent for m-PCR evaluation, as seen in Figure 2A, and was comparable to that of uniplex PCR (Figure 2B). Moreover, despite the presence of other bacterium sequences in the collection pool, only the DNA of these 8 viruses was replicated; no amplification happened with the interfering genomes. The sequencing findings confirmed the multiplex-PCR's high specificity.

### The m-PCR Technique's Efficiency

**Figure 3:**
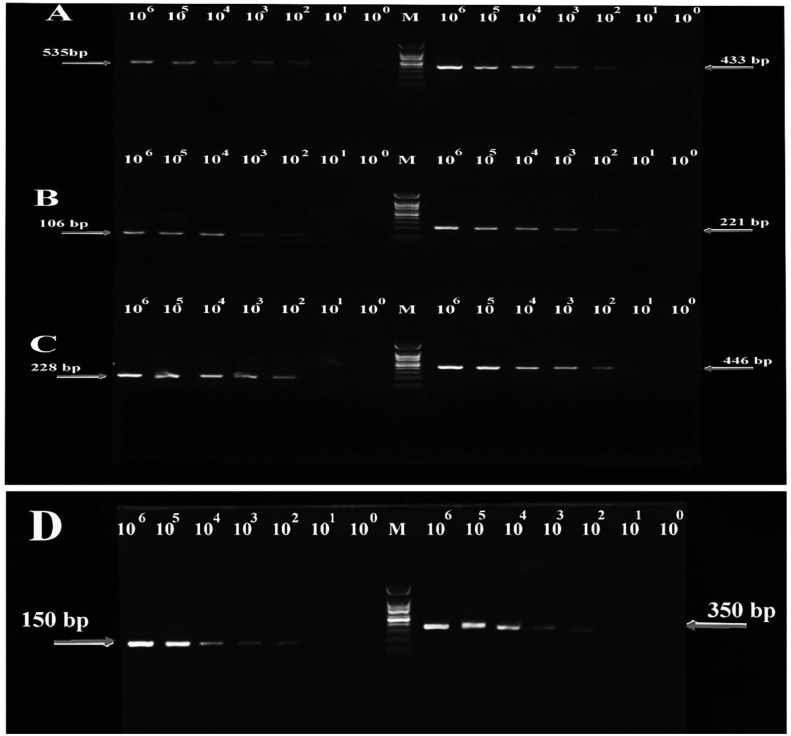
The m-PCR technique's efficiency. pcDNA3-NDV (A), pcDNA3-AIV (B), pcDNA3-IBV (C), and pcDNA3-IBDV (D) templates (d). M: stands for DNA ladder marker. Recombinant plasmids were diluted to a concentration of 10^8^ to 10^0^ DNA copies/μL. Gels are cropped to increase clarity and improve presentation conciseness. Samples were obtained from this experiment and the gels were processed in parallel. (10.6084/m9.figshare.20092625)

The m-PCR technique's efficiency for each viral sample was assayed by positive control (Recombinant plasmids). According to the results of Figure 3, the minimum detectable amount of virus in this kit is 100 (1 × 10^2^) copies. Therefore, the performance of this kit is equal to 100 copies of the number. In the next step, the kit was tested for verification with inactive virus samples.

### Sensitivity of the m-PCR Method

The lowest detectable concentration of viral genome is measured in this assay. The results demonstrated that this kit can detect the viral genome when the virus concentration in the sample is 100 copies per microliter. This kit's LOD value was 100 (1 × 10^2^ copies/μL) copies per microliter (Table 4).

**Table 4:** Sensitivity of kit by detection of LOD value.

Viral kit	Viral type	Serial 10-fold	Replicate1	Replicate2	Replicate3
NDV, AIV, IBV, IBDV genes	NDV- Class I	1 × 10^0^	-	-	-
		1 × 10^1^	-	-	-
		1 × 10^2^	+	+	+
		1 × 10^3^	+	+	+
		1 × 10^4^	+	+	+
		1 × 10^5^	+	+	+
		1 × 10^6^	+	+	+
	NDV- Class II	1 × 10^0^	-	-	-
		1 × 10^1^	-	-	-
		1 × 10^2^	+	+	+
		1 × 10^3^	+	+	+
		1 × 10^4^	+	+	+
		1 × 10^5^	+	+	+
		1 × 10^6^	+	+	+
	Avian influenza virus (AIV)	1 × 10^0^	-	-	-
		1 × 10^1^	-	-	-
		1 × 10^2^	+	+	+
		1 × 10^3^	+	+	+
		1 × 10^4^	+	+	+
		1 × 10^5^	+	+	+
		1 × 10^6^	+	+	+
	Avian influenza virus (AIV)	1 × 10^0^	-	-	-
		1 × 10^1^	-	-	-
		1 × 10^2^	+	+	+
		1 × 10^3^	+	+	+
		1 × 10^4^	+	+	+
		1 × 10^5^	+	+	+
		1 × 10^6^	+	+	+
	Infectious bronchitis virus	1 × 10^0^	-	-	-
		1 × 10^1^	-	-	-
		1 × 10^2^	+	+	+
		1 × 10^3^	+	+	+
		1 × 10^4^	+	+	+
		1 × 10^5^	+	+	+
		1 × 10^6^	+	+	+
	Infectious bronchitis virus (IBV-CK/CH/HUN/20180415)	1 × 10^0^	-	-	-
		1 × 10^1^	-	-	-
		1 × 10^2^	+	+	+
		1 × 10^3^	+	+	+
		1 × 10^4^	+	+	+
		1 × 10^5^	+	+	+
		1 × 10^6^	+	+	+
	IBDV(EGY-CK-IBDV-DAKH88-2021-VP2 VP2 gene, FJ20-9407)	1 × 10^0^	-	-	-
		1 × 10^1^	-	-	-
		1 × 10^2^	+	+	+
		1 × 10^3^	+	+	+
		1 × 10^4^	+	+	+
		1 × 10^5^	+	+	+
		1 × 10^6^	+	+	+
	IBDV(IR/H2965-17/18 VP2 gene,TN46/19)	1 × 10^0^	-	-	-
		1 × 10^1^	-	-	-
		1 × 10^2^	+	+	+
		1 × 10^3^	+	+	+
		1 × 10^4^	+	+	+
		1 × 10^5^	+	+	+
		1 × 10^6^	+	+	+

### Reproducibility of the m-PCR Method

Table 5 shows the findings of the m-PCR product's reproducibility. Multiplex-PCR was carried out using recombinant plasmids at ratios of 1 × 10^2^ copies/µL, showing the validity of the suggested approach. Two users repeated the test 20 times using kits made by a LOT and there was 100% concordance between the LOTs (Table 5). The investigation was also carried out 20 times by one user, using kits from 2 different LOTs. There was 100% agreement between the LOTs in the results of this test (Table S1).

**Table 5:** m-PCR technique NDV, AIV, IBV, IBDV kit reproducibility test.

Sample	Experimenter1				Experimenter2				Accordance rate (%)
	NDV	AIV	IBV	IBDV	NDV	AIV	IBV	IBDV	
1	+	+	+	+	+	+	+	+	100
2	+	+	+	+	+	+	+	+	100
3	+	+	+	+	+	+	+	+	100
4	+	+	+	+	+	+	+	+	100
5	+	+	+	+	+	+	+	+	100
6	+	+	+	+	+	+	+	+	100
7	+	+	+	+	+	+	+	+	100
8	+	+	+	+	+	+	+	+	100
9	+	+	+	+	+	+	+	+	100
10	+	+	+	+	+	+	+	+	100
11	+	+	+	+	+	+	+	+	100
12	+	+	+	+	+	+	+	+	100
13	+	+	+	+	+	+	+	+	100
14	+	+	+	+	+	+	+	+	100
15	+	+	+	+	+	+	+	+	100
16	+	+	+	+	+	+	+	+	100
17	+	+	+	+	+	+	+	+	100
18	+	+	+	+	+	+	+	+	100
19	+	+	+	+	+	+	+	+	100
20	+	+	+	+	+	+	+	+	100

### Result of Kit Validation

Multiplex-PCR tests were done in 3 different laboratories from different geographical location of Tehran to validate the kits manufactured in this work, and the findings obtained from the kits are 100% compatible with the results of the reference kit (GeneProof). Table 6 is used to display the information.

**Table 6:** Result of m-PCR kit validation using recombinant plasmids at ratios of 1 × 10^2^ copies/µL.

NDV, AIV, IBV, IBDV kit validation test									
Laboratory	Synthesized kit				GeneProof reference kit				Accordance rate (%)
	NDV	AIV	IBV	IBDV	NDV	AIV	IBV	IBDV	
AmitisGen	+	+	+	+	+	+	+	+	100
Bio3P	+	+	+	+	+	+	+	+	100
VetCare	+	+	+	+	+	+	+	+	100

### Model for Coinfection and Identification of Clinical Samples

Infections of various combinations of viruses were simulated at the same dose (1 × 10^2^ copies/µL). In addition, the developed m-PCR technology and uniplex PCR were used to investigate a total of 20 clinical specimens. Sixteen specimens tested positive for AIV, NDV, IBV, and IBDV. Six specimens were positive for AIV, 5 samples were positive for NDV, 3 samples were positive for IBV, and 2 samples were positive for IBDV. Table 7 shows the positive rate of each virus. Four of the positive samples had both NDV and AIV infections (Table 7). In addition, various experiments corroborated these findings. The results also revealed that sampling using live animal oral swabs was not substantially different from tissue sampling of a dead animal (*P* < 0.05), and this kit had a high sensitivity for evaluating both types of samples.

**Table 7:** Multiplex -PCR and uniplex PCR results for clinical positive samples.

NDV, AIV, IBV, IBDV kit										
Sample type	Sample *No.*	uniplex				Multiplex				Accordance rate (%)
		NDV	AIV	IBV	IBDV	NDV	AIV	IBV	IBDV	
Tissue specimens	1	-	+	-	-	-	+	-	-	100
	2	+	+	-	-	+	+	-	-	100
	3	-	-	-	+	-	-	-	+	100
	4	-	+	+	-	-	+	+	-	100
	5	+	+	-	-	+	+	-	-	100
	6	-	-	-	-	-	-	-	-	100
	7	+	-	+	-	+	-	+	-	100
	8	+	+	-	-	+	+	-	-	100
	9	-	-	+	-	-	-	+	-	100
	10	+	+	-	+	+	+	-	+	100
	Positive rates (%)	50%	60%	30%	20%	50%	60%	30%	20%	—
Oral swabs	1	-	+	-	-	-	+	-	-	100
	2	+	+	-	-	+	+	-	-	100
	3	-	-	-	+	-	-	-	+	100
	4	-	+	+	-	-	+	+	-	100
	5	+	+	-	-	+	+	-	-	100
	6	-	-	-	-	-	-	-	-	100
	7	+	-	+	-	+	-	+	-	100
	8	+	+	-	-	+	+	-	-	100
	9	-	-	+	-	-	-	+	-	100
	10	+	+	-	+	+	+	-	+	100
	Positive rates (%)	50%	60%	30%	20%	50%	60%	30%	20%	—

## DISCUSSION

Due to the general large financial damage caused by infection rates, increased mortality, and extensive medication tolerance, viral infectious disease remains a major concern in the poultry sector (Hafez and Attia, 2020). In recent decades, viral infectious disease in poultry has become more significant as a result of the growth of mixed culture systems, increased mobility of people and animals, and environmental contamination (Absalón et al., 2019). Additionally, the medical indications of numerous distinct viral diseases are quite similar, making it impossible to identify the agent without doing laboratory tests (Burrell et al., 2017). AIV, NDV, IBV, and IBDV are some of the most prevalent viral pathogens that affected poultry, posing substantial health risks and incurring significant economic losses (Dey et al., 2019). Virus isolation is the global standard for viral diagnosis; however, it is not appropriate for clinical fast identification (Piri Gharaghie et al., 2021). Different approaches for identifying such viral pathogens have included serological identification, immuno-electron scanning, ELISA, and real-time PCR (Absalón et al., 2019). Furthermore, there have not been any reports of the 8 viral pathogens being detected concurrently in poultry. As a result, we want to create a specific, sensitive, and quick m-PCR approach for diagnosing poultry viruses. Primers are the most important aspect in developing a successful multiplex-PCR process (Dronina et al., 2021). When various pairs of primers are combined, the number of interactions, such as mismatch and dimer, is prevalent (Álvarez-Fernández, 2013). An 8-plex PCR was designed as part of this study. The unique primers used in 8-plex PCR were found to be appropriate in this investigation. Following rigorous tuning, 8 pairs of viral primers were discovered, allowing each pathogen to be amplified individually. Furthermore, the amplification products matched the target gene segments. The results demonstrated that the m-PCR technique could flexibly identify the 8 viral pathogens and that a practical m-PCR technique had been effectively established. The amplifying efficiency can be influenced by a variety of different factors. Even the tiniest change in annealing temperature, for example, might cause nonspecific amplified (Rychlik et al., 1990). Furthermore, if the amount of Mg2+ in the process is very high, the approach lacks selectivity, and if it is excessively low, poor amplification is expected (Rychlik et al., 1990; Wang et al., 2019). As a result, we improved 4 performance factors, namely annealing temperature, Mg2+ quantity, Taq DNA polymerase concentration, and dNTP density, utilizing the design of experiments. The efficiency of an m-PCR experiment is usually decreased when the number of target genomes increased in the system (Wang et al., 2019). The detection limit of the suggested m-PCR test, on the other hand, was 1 × 10^2^ copies/µL of each virus species, which is consistent with earlier research. The efficiency of the suggested m-PCR technique for detecting *E. coli* was 10^3^ CFU/mL, which was higher than the 10^4^ CFU/mL reported by Kong et al. (2002). AIV, NDV, IBV, IBDV, and other viruses and bacteria that may infect poultry were used to test the m-PCR technique's specificity. When all of the DNA precursors were available in the test pool, the m-PCR approach produced no crossing interactions between these viruses or nonspecific responses with other frequent poultry microorganisms (Liu et al., 2018), suggesting that the designed primers were extremely specialized. Moreover, it is usual in clinical practice to have many infections infected at the same time. Whenever poultry become infected with one viral pathogen, they become vulnerable to others (Zhang et al., 2009; Shen et al., 2016), which may have a stronger virulence and result in greater financial damage (Doosti et al., 2009; Gao et al., 2014). As a result, coinfection was used to test the viability of the m-PCR approach. The results of the coinfection research were compatible with the classification of 8 viral pathogens, demonstrating that the m-PCR approach could recognize numerous random viral mixtures flexibly and specifically. The m-PCR approach had a sensitivity limit of 1 × 10^2^ copies/μL, which was similar to the uniplex PCR approach. The m-PCR approach, on the other hand, is more useful due to its ease of use and maximum throughput in laboratory testing (Kargar et al., 2012; Junlong et al., 2015). Furthermore, the m-PCR approach produced extremely consistent findings when used with uniplex PCR, proving the technology's precision. The assay was carried out to determine the technique's repeatability and reproducibility. This indicated that the m-PCR approach was extremely dependable and stable. The results of the detection and quantification using the newly designed m-PCR technique were comparable to those obtained using the uniplex PCR and conventional PCR methods. Sequencing was also used to verify all clinically positive results. The samples taken from farm products did not produce any distinct band, and the positive samples were from a variety of poultry farms. As a result, the samples taken from the marketplaces were both healthy and safe. In addition, this finding revealed that the m-PCR approach was specific, sensitive, quick, and useful in laboratory and clinical diagnostics.

We developed an m-PCR approach that could identify and discriminate 8 main viruses that cause poultry illnesses, including AIV, NDV, IBV, and IBDV, with excellent specificity, sensitivity, and repeatability. As a result, preventative measures may be put in place as soon as feasible to minimize financial damage.

## Appendix Supplementary materials

Image, application 1

## Footnotes

Supplementary material associated with this article can be found in the online version at doi:10.1016/j.psj.2023.102852.
