# Supplementary material for: Simultaneous and rapid detection of avian respiratory diseases of small poultry using multiplex reverse transcription-Polymerase Chain Reaction assay
Source: Poult Sci. 2023 Jun 8;102(8):102852. doi: 10.1016/j.psj.2023.102852 (PMC10404739; doi:10.1016/j.psj.2023.102852)
Supplement: Supplementary file 1 [file mmc1.docx]

**supplementary files**

**Table S1** Multiplex-PCR technique repeatability

| ****NDV, AIV, IBV, IBDV**** kit repeatability test | | | | | | | | | |
| --- | --- | --- | --- | --- | --- | --- | --- | --- | --- |
| Sample NO. | ***LOT1*** | | | | **LOT2** | | | | **Accordance rate (%)** |
|  | **NDV** | **AIV** | **IBV** | **IBDV** | **NDV** | **AIV** | **IBV** | **IBDV** |  |
| 1 | + | + | + | + | + | + | + | + | 100 |
| 2 | + | + | + | + | + | + | + | + | 100 |
| 3 | + | + | + | + | + | + | + | + | 100 |
| 4 | + | + | + | + | + | + | + | + | 100 |
| 5 | + | + | + | + | + | + | + | + | 100 |
| 6 | + | + | + | + | + | + | + | + | 100 |
| 7 | + | + | + | + | + | + | + | + | 100 |
| 8 | + | + | + | + | + | + | + | + | 100 |
| 9 | + | + | + | + | + | + | + | + | 100 |
| 10 | + | + | + | + | + | + | + | + | 100 |
| 11 | + | + | + | + | + | + | + | + | 100 |
| 12 | + | + | + | + | + | + | + | + | 100 |
| 13 | + | + | + | + | + | + | + | + | 100 |
| 14 | + | + | + | + | + | + | + | + | 100 |
| 15 | + | + | + | + | + | + | + | + | 100 |
| 16 | + | + | + | + | + | + | + | + | 100 |
| 17 | + | + | + | + | + | + | + | + | 100 |
| 18 | + | + | + | + | + | + | + | + | 100 |
| 19 | + | + | + | + | + | + | + | + | 100 |
| 20 | + | + | + | + | + | + | + | + | 100 |
